# Liquid biopsy: from concept to clinical application

**DOI:** 10.1038/s41598-023-48501-x

**Published:** 2023-12-07

**Authors:** Catherine Alix-Panabières, Dario Marchetti, Julie E. Lang

**Affiliations:** 1 Laboratory of Rare Circulating Human Cells (LCCRH), University Medical Center of Montpellier, Montpellier, France; 2 CREEC/CANECEV, MIVEGEC (CREES), Université de Montpellier, CNRS, IRD, Montpellier, France; 3 European Liquid Biopsy Society (ELBS), Hamburg, Germany; 4 Departments of Internal Medicine and Pathology, The University of New Mexico Health Sciences Center, UNM Comprehensive Cancer Center, MSC07 4025, 1 University of New Mexico, Albuquerque, NM 87131 USA; 5 Breast Surgery and Cancer Biology, Cleveland Clinic, 9500 Euclid Ave, A80, Cleveland, OH 44195 USA; 6 Case Comprehensive Cancer Center, Cleveland Clinic, 9500 Euclid Ave, A80, Cleveland, OH 44195 USA; 7 Institut Universitaire de Recherche Clinique (IURC), 641, avenue du Doyen Gaston Giraud, 34093 Montpellier Cedex 5, France

**Keywords:** Metastasis, Biomarkers

## Abstract

The diagnosis and treatment of cancer presents a physical and mental burden to the patient, often involving diagnostic biopsies and surgeries or chemotherapeutic approaches with severe side-effects. Advances which enable early detection of cancer and close monitoring of the disease course without invasive procedures, and which can underpin a tailored approach to treatment, can therefore make a big difference to the quality of life of patients. Liquid biopsies can be used to access tumor cells and tumor DNA circulating in the blood. Monitoring these species can provide a minimally invasive and repeatable means to detect cancer, or gain information about its response to treatment.

The concept of liquid biopsy, as a minimally invasive blood sample collected throughout the course of disease, was first introduced (for circulating tumor cells, or CTCs) in 2010 and consistently applied afterwards^1^. To obtain a comprehensive real-time view of cancer progression, we must consider the broader definition of liquid biopsy which includes: (i) other tumor-derived circulating biomarkers such as circulating cell-free tumor DNA (ctDNA), circulating cell-free RNA (noncoding, miRNA and messenger RNA), extracellular vesicles (exosomes and oncosomes), tumor-educated platelets, and circulatory proteins; and (ii) circulating immune cells and immune system components. Furthermore, the term liquid biopsy in 2023 can also include the identification of the circulating microbiome in the blood, coined liquid microbiopsy, which is done by analyzing the circulating cell-free microbial DNA in combination with a defined panel of proteins and metabolites^2^. Lastly, this concept has been extended to other physiological fluids such as cerebrospinal fluid, urine, bone marrow, sputum, and saliva^3^.

Clinically, liquid biopsy can be used for: (i) early detection of cancer using high blood volumes, though screening remains a challenge; (ii) tumor staging and monitoring of patients with localized cancer, e.g. to distinguish patients at low and high risk of recurrence; (iii) predicting metastatic progression in patients with advanced cancer; and (iv) monitoring treatment efficacy.

This collection on current advances of liquid biopsy research includes articles written by experts in this field of research and covers multiple facets of liquid biopsy.

## CTCs

Minimal residual disease detection by liquid biopsy has been shown to allow for the identification of metastatic disease as much as 2 years earlier than by imaging^4,5^. Stergiopoulou et al. provide evidence that liquid biopsy can detect minimal residual disease in breast cancer^6^. The authors enumerated and analyzed CTCs at the phenotypical and molecular levels (proteins, gene expression, epigenetic). Asante et al. worked on high grade serous ovarian carcinoma and reported the importance of genetic analysis of heterogenous subsets of CTCs to confirm their neoplastic origin^7^. This study was carried out only on 4 patients and the data obtained certainly need to be confirmed on a larger number of patient cohorts. Bao-Caamano et al. focused on performing epigenomic analyses of metastasis-competent colon cancer CTCs and showed that they present a unique DNA methylation program^8^. This study provides new insights into the epigenomic landscape of such aggressive subset of CTCs, revealing biological information for metastasis development, as well as cues for new potential biomarkers and therapeutic targets for colorectal cancer. Finally, Pirone et al. presented a label‑free liquid biopsy through the identification of CTCs by machine learning‑powered tomographic phase imaging flow cytometry^9^.

## CtDNA

Calapre et al. compared the suitability of the Accel (Swift) and Oncomine (ThermoFisher Scientific, Inc.) panels for identification of TP53 mutations in ctDNA of 10 high grade serous ovarian carcinoma patients, and concluded that the Oncomine panel possessing unique molecular identifiers appears more useful^10^ for ctDNA analysis. Thus, this study demonstrates the utility of unique molecular identifiers-tagged NGS panel for plasma TP53 mutation screening in these patients.

## Circulating cell-free miRNA

Gahlawat et al. reported that circulating cell-free miRNA can be more easily obtained as a very stable biomarker population in blood, thus it might serve as a more appropriate surrogate liquid biopsy marker than cfDNA for ovarian cancer^11^.

## Circulating cell-free mRNA

Grosgeorges et al. selected 3 candidates for colorectal cancer (B2M, TIMP-1, and CLU) and developed a method to purify circulating cell-free mRNAs from plasma samples by quantifying them via RT-qPCR^12^. Based upon these findings, this new approach was able to discriminate metastatic colorectal cancer patients from healthy donors.

## Non-coding DNA

Baxter et al. reported that specific hotspots in non-coding regions of ADGRG6, PLEKHS1, WDR74, TBC1D12 and LEPROTL1 could function as liquid biopsy in the urine for non-invasive detection of bladder cancer^13^. In the absence of clinically detectable disease during surveillance, the presence of non-coding mutations in urine was associated with an increased relative risk of future recurrence.

## Exosomes

Shaikh et al. investigated the expression levels of a specific gene panel in exosomes isolated from patients with oral cancer to lymph nodes metastasis along with an integrated computational screening^14^. Their significant gene signature identification demonstrated increased serum exosome efficacy in early detection and was clinically associated with intracellular communication in the formation of the premetastatic niche.

In conclusion, liquid biopsy analyses can be used to gain new insights into the biology of metastasis, its prediction and/or prevention, to be a companion diagnostic to improve therapy stratification, and to gain insights into therapy-induced cancer cell selection. Within these contexts, intra-patient tumor heterogeneity may be an important mechanism to investigate for total eradication of all tumor clones, including but also extending CTC subsets, by targeted therapies^15^. In addition, the synergy of multiple circulating biomarkers can reveal the molecular specifics of cancer^16^.

Researchers and clinicians have been aware for many years of the potential value of liquid biopsies as useful tools to complement current therapies non-invasively and in real-time in the patients^17,18^. Liquid biopsy has also been implemented in clinical trials to measure biomarkers indicative of treatment response^19^ and prognosis^20–22^, meanwhile observational studies demonstrated the clinical utility of liquid biopsy to predict therapeutic response before it is clinically apparent^23–25^ and to better understand the biology of tumors^26^. Personalized mutation tracking using custom-made bespoke assays have also been designed for non-metastatic patients to allow for a more accurate screening for disease recurrence while patients are potentially curable^27^.

More interventional clinical trials are urgently needed to widely implement liquid biopsy in clinical practice. Policymakers and business leaders must participate in these clinical trials and discussions in order to make national and international decisions. Of particular significance, the multi-center standardization of preanalytical and analytical methods is imperative before liquid biopsy can be consistently used in clinical settings. Big consortia such as the European Liquid Biopsy Society (ELBS; www.elbs.eu) in the EU, or the BLOODPAC in the U.S.A. continue to lead significant programs to complete this mission and develop and validate a wide range of standard operating procedures. There is also a clinically unmet need for more studies of liquid biopsy approaches to diagnose cancer before it is clinically evident, particularly for those types of cancer in which there is no clinical screening testing available^28^. 

Multidisciplinary collaboration between academia, the biotechnology and pharmaceutical industries, and other stakeholders will be crucial in moving the field forward. We anticipate that precision medicine approaches that identify high-risk patient populations and predict specific therapeutic benefit for patients will ultimately succeed in gaining clinical traction as the field moves beyond simply enumerating the presence of minimal residual disease to incorporating liquid biopsy in well-designed clinical trials in cancer.

Finally, this collection of articles highlights the diversity of liquid biopsy approaches and the many strengths afforded by each strategy. Many challenges still remain to make liquid biopsy a reality in the clinic. We hope it will inspire researchers to continue innovating the applications of liquid biopsy and making important discoveries as we work together towards the goal of translating liquid biopsy to the clinic to improve outcomes for cancer patients.
